# Health Tract, No. 150

**Published:** 1863-07

**Authors:** 


HEALTH TRACT, No. 150.

### LOOSE BOWELS.

THERE are three kinds of loose bowels, technically called "diarrhea," or a "flowing through" of water, bile, or blood. If it is water, it is *diarrhea* proper; if it is bile, it is *bilious diarrhea*: if it is blood, it is *dysentery*. Simple diarrhea is a thin, light-colored discharge from the bowels, occurring five, ten, or twenty times in twenty-four hours; if let alone it becomes Asiatic cholera in certain states of the atmosphere. Its great characteristic is the extraordinary debilitating effect which speedily pervades the whole body; the patient feels, when he sits down, as if it would be a happiness just to be allowed to remain there. Absolute quietude is an elysium to him. Instinct calls for the most perfect rest possible, and thus points out the most certain and appropriate of all modes of cure, which is absolute and continuous rest on a bed, in a cool, clean, well-aired room, until the passages assume the consistency of mason's mortar, and not oftener than twice in twenty-four hours. In health the bowels are incessantly moving, not unlike worms in a carrion; hence the ancients designated it as the "vermicular action," *vermis* meaning a worm. If there is not activity enough, we have constipation, or torpid, *sleepy* action; when this action is excessive, it is diarrhea. Every step a man takes has a tendency to set the bowels in motion; hence one of the most certain and frequent and efficient cures of constipation, when the bowels act but once in two or three or more days, is to be moving about on the feet almost all the time. If then motion tends to increase the activity of the bowels, when that activity is too great, instinct, alike with reason, dictates as perfect quietude as possible. If the symptoms do not abate by simply resting on a bed, a greater quietude of the vermicular motion is compelled by simply binding a strip of woolen flannel, about fourteen inches wide, tightly around the abdomen or "stomach," so as to be double in front, the effect of which is to give the bowels less room to move about in; affords remarkable strength to the whole body, and keeps the surface warm, soft, and moist. As the disease is a too great flow of fluids through the system, drinking fluids of any description only aggravates the malady. Yet, as the thirst is sometimes excessive, lumps of ice may be chewed and swallowed in as large pieces as possible, to any extent desired. No food should be eaten except rice, parched like coffee, boiled as usual, served, and eaten with an equal bulk of boiled milk. This may be varied by boiling a pint of flour in a linen bag, in milk, for an hour or two, skin off the outside, dry it, grate it in boiled milk, make it palatable with salt or sugar, and eat as much as desired every fifth hour during the day, eating and drinking nothing else. This treatment will cure nine cases out of ten, if adopted promptly within forty-eight hours; if not, call in a physician.

